# Clinical features that predict the mortality risk in older patients with Omicron pneumonia: the MLWAP score

**DOI:** 10.1007/s11739-023-03506-2

**Published:** 2023-12-16

**Authors:** Yongjian Pei, Ting Li, Chen Chen, Yongkang Huang, Yun Yang, Tong Zhou, Minhua Shi

**Affiliations:** Department of Respiratory and Critical Care Medicine, The Second Affiliated Hospital of Soochow University, 1055 SanXiang Road, Gusu District, Suzhou, 215004 Jiangsu China

**Keywords:** Omicron pneumonia, SARS-CoV-2, Predicting mortality, Predictive score model

## Abstract

In December 2022, the Chinese suffered widespread Omicron of SARS-CoV-2 with variable symptom severity and outcome. We wanted to develop a scoring model to predict the mortality risk of older Omicron pneumonia patients by analyzing admission data. We enrolled 227 Omicron pneumonia patients aged 60 years and older, admitted to our hospital from December 15, 2022, to January 16, 2023, and divided them randomly into a 70% training set and a 30% test set. The former were used to identify predictors and develop a model, the latter to verify the model, using the area under the receiver operating characteristic curve (AUC), the Hosmer–Lemeshow goodness-of-fit test, a calibration curve to test its performance and comparing it to the existing scores. The MLWAP score was calculated based on a multivariate logistic regression model to predict mortality with a weighted score that included immunosuppression, lactate ≥ 2.4, white blood cell count ≥ 6.70 × 10^9^/L, age ≥ 77 years, and PaO_2_/FiO_2 _≤ 211. The AUC for the model in the training and test sets was 0.852 (95% CI, 0.792–0.912) and 0.875 (95% CI, 0.789–0.961), respectively. The calibration curves showed a good fit. We grouped the risk scores into low (score 0–7 points), medium (8–10 points), and high (11–13 points). This model had a sensitivity of 0.849, specificity of 0.714, and better predictive ability than the CURB-65 and PSI scores (AUROC = 0.859 *vs*. 0.788 *vs*. 0.801, respectively). The MLWAP-mortality score may help clinicians to stratify hospitalized older Omicron pneumonia patients into relevant risk categories, rationally allocate medical resources, and reduce the mortality.

**Supplementary Information:**

The online version contains supplementary material available at 10.1007/s11739-023-03506-2.

## Introduction

COVID-19 is an acute respiratory infectious disease caused by a new coronavirus type 2 (SARS-CoV-2) that is characterized by fever, fatigue, and a dry cough. In severe cases, it can develop rapidly into acute respiratory distress syndrome, septic shock, metabolic acidosis, and coagulation dysfunction [1]. SARS-CoV-2 is evolving, and new Alpha, Beta, Gamma, and Delta variants have emerged since the onset of COVID-19 [2]. Since January 2022, the Omicron variant has surpassed all previous mutants in its super transmissibility and immune escape and replaced Delta as the principal transmission strain in the world [3]. In December 2022, a large outbreak of the Omicron variant occurred in China. Despite indications that its clinical severity may be lower than that of Delta [4], the sudden surge of Omicron pneumonia patients placed unprecedented pressure on the Chinese healthcare system [5].

Additionally, there are many variations of the Omicron variant [6], with heterogeneity in the severity of the illness and the possibility of reinfection [7, 8]. By the end of June 2023, over 690 million people worldwide had been infected with COVID-19, causing nearly 6.9 million deaths. Reducing the mortality of critically ill patients requires early identification and medical intervention. Identifying the severity of the disease or patients at high-risk of death is a critical step in managing COVID-19. There are multiple tools for predicting the prognosis of pneumonia clinically, such as CRB-65, CURB-65, and PSI, which are widely used for evaluating community-acquired pneumonia (CAP) [9]. However, most are still not applicable for viral infections, such as COVID-19. Many scoring systems based on other diseases are used for assessing the adverse prognosis risk of COVID-19, including commonly used scores for CAP, viral pneumonia, and early sepsis recognition [10]. Age is positively associated with the severity and mortality of COVID-19 infection [11]. To our knowledge, there are no severity scoring methods for Omicron pneumonia in older adults.

Here, we assessed the clinical and laboratory characteristics of older Omicron pneumonia patients on admission and developed a new scoring system to predict their mortality risk.

## Materials and methods

### Study design and population

We retrospectively studied all hospitalized patients aged 60 years and older with a positive real-time polymerase chain reaction (RT-PCR; TIB respiratory kit, ROCHE, Switzerland) test for SARS-CoV-2 on nasopharyngeal swabs and no previous SARS-CoV-2 infections, who were admitted to the Second Affiliated Hospital of Soochow University, China, from December 15, 2022, to January 16, 2023 (during the rapid spread of the Omicron variant in China). The study was approved by the hospital ethics committee (no. JD-LK2023025-I01, approved in March 2023) and the need for informed consent was waived as it was a retrospective analysis. Our study enrolled patients diagnosed with pneumonia based on the 2009 Infectious Diseases Society of America (IDSA)/American Thoracic Society (ATS) criteria [12]. Following World Health Organization standards, Omicron pneumonia was divided into non-severe, severe, and critically ill [13]. The Omicron pneumonia patients included in this study had comorbid immunosuppression, including tumor patients undergoing radiation or chemotherapy, long-term use of steroids or immunosuppressive drugs for autoimmune diseases, and lymphocyte counts < 0.8 × 10^9^/L. Exclusion criteria were age < 60 years, those ultimately diagnosed with other non-pneumonia diseases, and those hospitalized for more than 3 months before death.

**Fig. 1:**
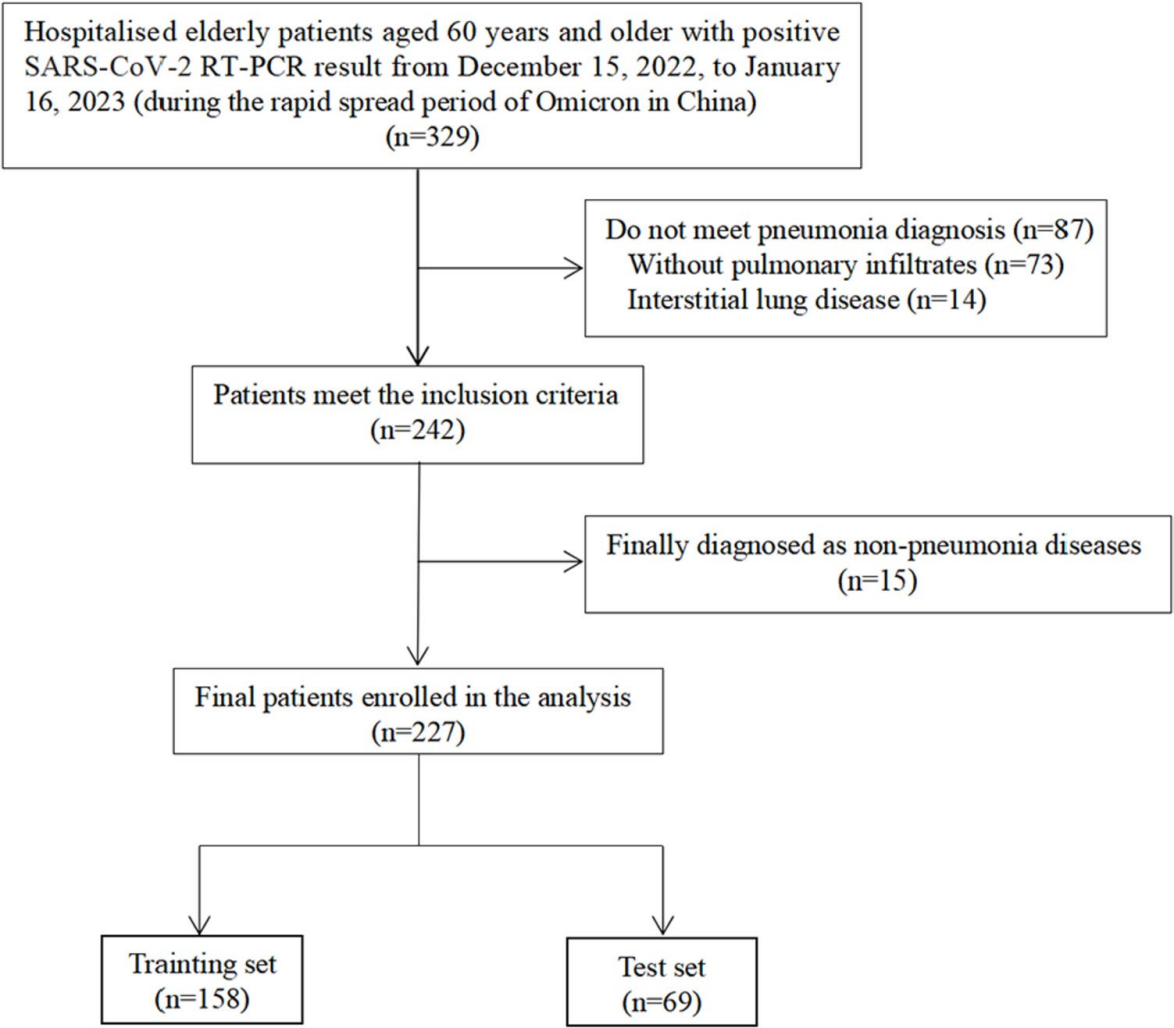
Study flowchart. RT-PCR: reverse-transcription polymerase chain reaction

Initially, 329 hospitalized patients aged 60 years or older had positive RT-PCR tests for SARS-CoV-2 on nasopharyngeal swabs, and 242 of them with pneumonia were enrolled. From these, 15 cases diagnosed with other non-pneumonia diseases were excluded (*n* = 15), leaving 227 pneumonia patients with positive RT-PCR testing for SARS-CoV-2 on nasopharyngeal swabs in the analysis. This population was randomly divided on a 70:30 basis into training and test sets. The training set was used to identify predictors and develop a multivariate model. The test set was used to assess the discrimination and calibration of the model (Fig. 1).

### Data collection

Infection caused by SARS-CoV-2 was confirmed by RT-PCR detection on nasopharyngeal swabs. We collected the patients’ admission data, including demographic information, comorbidities, symptoms, number of days before admission symptoms began, laboratory examinations, chest x-ray or CT images, respiratory tract secondary infection, treatment options, complications, intensive care unit (ICU) admission, and invasive mechanical ventilation. Myocardial injury was defined as a new elevation of the cardiac troponin (cTn) level exceeding the 99th percentile of the upper reference limit [14]; our reference range for cTn is 0–30 pg/mL. Acute renal injury refers to an increase in the serum creatinine (sCr) level by at least 26.5 μmol/L (0.3 mg/dL) within 48 h, an increase of 1.5 times the baseline sCr value within 7 days, or a decrease in urine output to 0.5 mL/kg/h for at least 6 h [15]. We calculated the CURB-65 [9] and PSI [16] scores of all patients and recorded their outcomes. Mortality was defined as the total mortality within 90 days.

### Statistical methods

We randomly divided the 227 older adult Omicron pneumonia patients into a training set of 158 (70%) and a test set of 69 (30%), and classified the training set into survivor and 90-day death groups. The statistical analyses were done using SPSS ver. 26.0 (IBM, Armonk, NY, USA) and R4.2.3 (www.r-project.org). For continuous variables that conformed to a normal distribution, groups were compared using the *t*-test and reported as the mean ± standard deviation; otherwise, the non-parametric *U*-test was used and the results as the median (interquartile range). Enumeration data are expressed as n (%), and groups were compared using Pearson’s chi-square test. Taking the 90-day death as the dependent variable, variables with a *p*-value less than 0.05 in univariate analyses were used as independent variables, using a backward conditional method to obtain a multivariate logistic regression model in the training set, using the odds ratios (OR) as the risk assessment parameter. The performance of the score was evaluated by measuring the area under the ROC curve (AUROC), and the sensitivity and specificity were calculated. A calibration curve and the Hosmer–Lemeshow goodness-of-fit test were used to assess the calibration. The calibration of the model in both the training and test sets was validated using 1000 bootstrap resamplings. The points for each predictor were summed to calculate the total risk score for each patient. We grouped the risk scores into three categories (low-, medium-, and high-risk of 90-day mortality) and plotted the observed versus predicted proportion of 90-day mortality of the patients in each category in the test set. We used the ROC curve to assess the risk prediction ability of the score compared with the CURB-65 and PSI scores. All tests were two-sided and a *p*-value < 0.05 was considered a significant statistical difference.

## Results

### Demographics and clinical features

Our study included 227 older adult Omicron pneumonia patients of whom 32.2% (73/227) died. We randomly divided the entire sample into a training set with 158 patients and a test set with 69 patients (Fig. 1). Table S1 summarizes the clinical features of the two cohorts. Table 1 compares the demographics and baseline characteristics between the survivors and those who died within 90-days after admission in the training set. The survivor group comprised 105 (66.5%) patients, while the 90-day death group comprised 53 (33.5%) patients. The training sample comprised 102 (64.56%) males and 56 (35.44%) females for a gender ratio of 1.8. The median age of the 158 cases was 77 years, and the survival and 90-day death group was 74 years and 81 years, respectively. The two groups did not differ significantly in body mass index (BMI). The most common comorbidity was cardiovascular disease (70.25%), followed by immunosuppression, diabetes, malignant tumor, cerebrovascular disease, and chronic lung disease. The 90-day death group had more comorbid immunosuppression and cerebrovascular disease than the survivor group, and both differed statistically (*p* < 0.05). The most common symptoms were cough and fever, followed by chest tightness and pharyngalgia. The survivor group had more fever symptoms than the 90-day death group; while the opposite was true for chest tightness (*p* < 0.05). In the 90-day death group, 94.34% (50/53) of the cases had critical COVID-19 pneumonia, 56.60% (30/53) were admitted to the ICU, and 35.85% (19/53) received invasive mechanical ventilation, all significantly higher than in the survivor group, and were treated significantly more often with sivelestat than the survivor group (*p* < 0.05). There were no statistical differences in administering antiviral agents, anticoagulation, baricitinib, tocilizumab, human immunoglobulin, or glucocorticoid between the two groups. In terms of complications, the 90-day death group had significantly more respiratory tract secondary infection, myocardial injury, and acute kidney injury (AKI) than the survivor group (all *p* < 0.05).

**Table 1:** Comparisons of demographics and baseline characteristics between survivors and those who died in 90-days after admission of the training set

Variable	Total (*n* = 158)	Survivors (*n* = 105)	Died (*n* = 53)	χ2/Z	*p*-value^※^
Male	102 (64.56)	68 (64.76)	34 (64.15)	0.006	0.940
Age (years)	77.00 (70.00–84.00)	74.00 (67.00–81.00)	81.00 (74.00–86.00)	3.748	** < 0.001**
BMI (kg/m^2^)	23.00 (21.00–25.00)	23.00 (21.00–25.00)	23.00 (20.00–24.00)	1.440	0.153
Chronic lung disease	17 (10.76)	11 (10.48)	6 (11.32)	0.026	0.871
Cardiovascular disease	111 (70.25)	74 (70.48)	37 (69.81)	0.007	0.931
Cerebrovascular disease	23 (14.56)	10 (9.52)	13 (24.53)	6.375	**0.012**
Diabetes	57 (36.08)	39 (37.14)	18 (33.96)	0.155	0.694
Immunosuppression	92 (58.23)	53 (50.48)	39 (73.58)	7.733	**0.005**
Malignant tumor	31 (19.62)	23 (21.90)	8 (15.09)	1.036	0.309
Days of onset of symptoms before admission	7.00 (4.00–10.00)	7.00 (5.00–10.00)	7.00 (3.00–9.00)	0.785	0.439
Fever	110 (69.62)	79 (75.24)	31 (58.49)	4.671	**0.031**
Cough	113 (71.52)	77 (73.33)	36 (67.92)	0.506	0.477
Chest tightness	81 (51.27)	47 (44.76)	34 (64.15)	5.300	**0.021**
Pharyngalgia	17 (10.76)	13 (12.38)	4 (7.55)	0.857	0.355
Multi-lobular infiltration	129 (81.65)	85 (80.95)	44 (83.02)	0.100	0.751
CURB 65 score	2.00 (1.00–3.00)	2.00 (1.00–2.00)	3.00 (2.00–3.00)	6.334	** < 0.001**
PSI score	107.50 (91.00–128.00)	99.00 (82.00–115.00)	126.00 (104.00–152.00)	5.411	** < 0.001**
Omicron pneumonia				121.258	** < 0.001**
Non-severe	48 (30.38)	48 (45.71)	0 (0.00)		
Severe	54 (34.18)	51 (48.57)	3 (5.66)		
Critical	56 (35.44)	6 (5.71)	50 (94.34)		
PaO_2_/FiO_2_	211.00 (123.00–312.00)	265.00 (202.00–345.00)	96.00 (68.00–153.00)	8.398	** < 0.001**
Lac	2.40 (1.50–4.07)	1.90 (1.40–2.60)	4.20 (2.80–5.70)	6.603	** < 0.001**
White blood cell counts, × 10^9^/L [4–10]	6.70 (4.70–9.88)	6.00 (4.30–8.00)	9.40 (6.00–12.30)	4.595	** < 0.001**
Neutrophil counts, × 10^9^/L [1.8–6.3]	5.20 (3.40–8.70)	4.25 (3.10–6.23)	8.70 (5.20–11.50)	5.644	** < 0.001**
Lymphocyte counts, × 10^9^/L [1.1–3.2]	0.70 (0.50–1.00)	0.80 (0.50–1.20)	0.60 (0.30–0.80)	3.828	** < 0.001**
CRP (mg/L)	63.00 (28.00–140.00)	43.60 (16.60–86.43)	145.60 (75.20–231.40)	5.839	** < 0.001**
PCT(ng/mL)	0.15 (0.06–0.76)	0.10 (0.05–0.25)	0.49 (0.12–4.65)	4.618	** < 0.001**
IL-6 (pg/mL)	20.23 (6.37–56.88)	15.49 (2.12–36.07)	54.00 (18.00–110.30)	4.866	** < 0.001**
D- dimer (μg/mL)	2.15 (0.93–6.00)	1.35 (0.69–2.66)	6.57 (4.13–12.66)	6.780	** < 0.001**
Admitted to ICU	42 (26.58)	12 (11.43)	30 (56.60)	36.831	** < 0.001**
Invasive mechanical ventilation	21 (13.29)	2 (1.90)	19 (35.85)	35.214	** < 0.001**
Respiratory tract secondary infection	40 (25.32)	16 (15.24)	24 (45.28)	16.816	** < 0.001**
Myocardial injury	67 (42.41)	29 (27.62)	38 (71.70)	28.020	** < 0.001**
AKI	53 (33.54)	21 (20.00)	32 (60.38)	25.759	** < 0.001**
Hepatic insufficiency	76 (48.10)	49 (46.67)	27 (50.94)	0.258	0.611
Antiviral	99 (62.66)	69 (65.71)	30 (56.60)	1.249	0.264
Sivelestat	23 (14.56)	7 (6.67)	16 (30.19)	15.668	** < 0.001**
Baricitinib	11 (6.96)	8 (7.62)	3 (5.66)	0.016	0.900
Tocilizumab	10 (6.33)	4 (3.81)	6 (11.32)	2.205	0.138
Human immunoglobulin	25 (15.82)	13 (12.38)	12 (22.64)	2.784	0.095
Glucocorticoid	143 (90.51)	97 (92.38)	46 (86.79)	1.280	0.258

Continuous variables presented as median (upper and lower quartiles), enumeration data as *n* (%). All clinical characteristics were collected on admission. ^※^*P*-value represented the comparison between survival group and death group. The bolded values are *p*-values < 0.05, which indicates the significant difference between the two groups. *BMI* body mass index, *Lac* lactate, *COVID* coronavirus disease, *CRP* C-reactive protein, *PCT* procalcitonin, *IL-6* Interleukin 6, *ICU* intensive care unit, *AKI* acute kidney injury

### Laboratory findings

The patients underwent laboratory testing on the day of admission (Table 1). In the training set, the CURB-65 and PSI scores and proportion of critical Omicron pneumonia were significantly higher in the 90-day death group than in the survivor group. In the 90-day death group, the PO_2_/FiO_2_ and lymphocyte counts were lower than in the survivor group (*p* < 0.001). Moreover, the lactate (Lac) content, white blood cell and neutrophil counts, C-reactive protein (CRP), procalcitonin (PCT), Interleukin 6 (IL-6), and d-dimer were all higher in the 90-day death group than in the survivor group (all *p* < 0.001).

### Risk factors of mortality

**Fig. 2:**
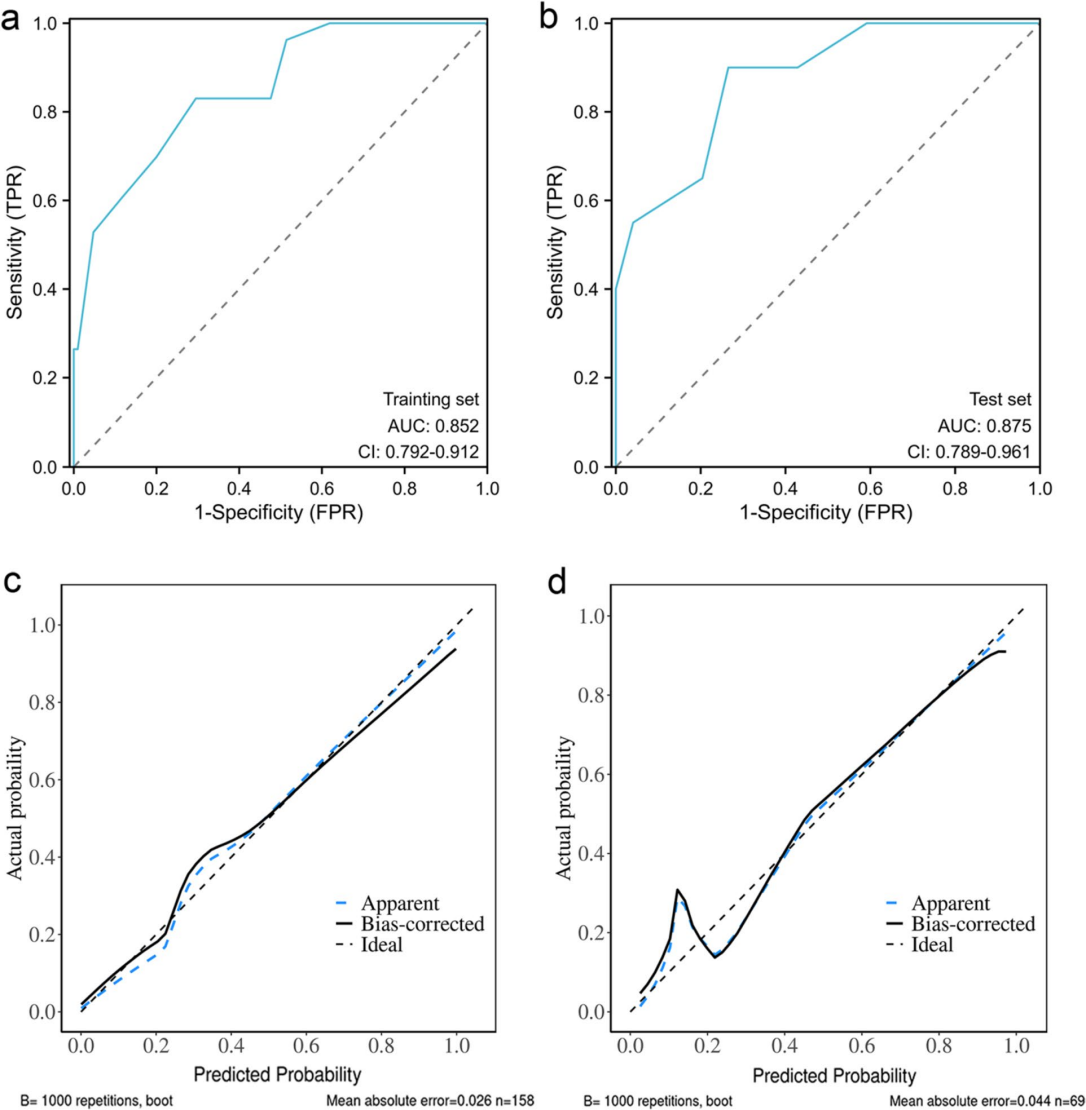
The receiver operating characteristic curve and calibration curve. (**a**) AUC curve of the training set; (**b**) AUC curve of the test set; (**c**) Calibration plot for the training set with bootstrapping; (**d**) Calibration plot of the test set with bootstrapping. In (**c**) and (**d**), X-axis is the predicted probability of 90-day death risk; Y-axis is the actually observed probability. The dashed gray line indicates a perfect prediction by an ideal model. The blue solid line represents the calibration of prediction model, while black solid line is bias-corrected with bootstrapping technique. *AUC* area under the receiver operating characteristic curve

**Fig. 3:**
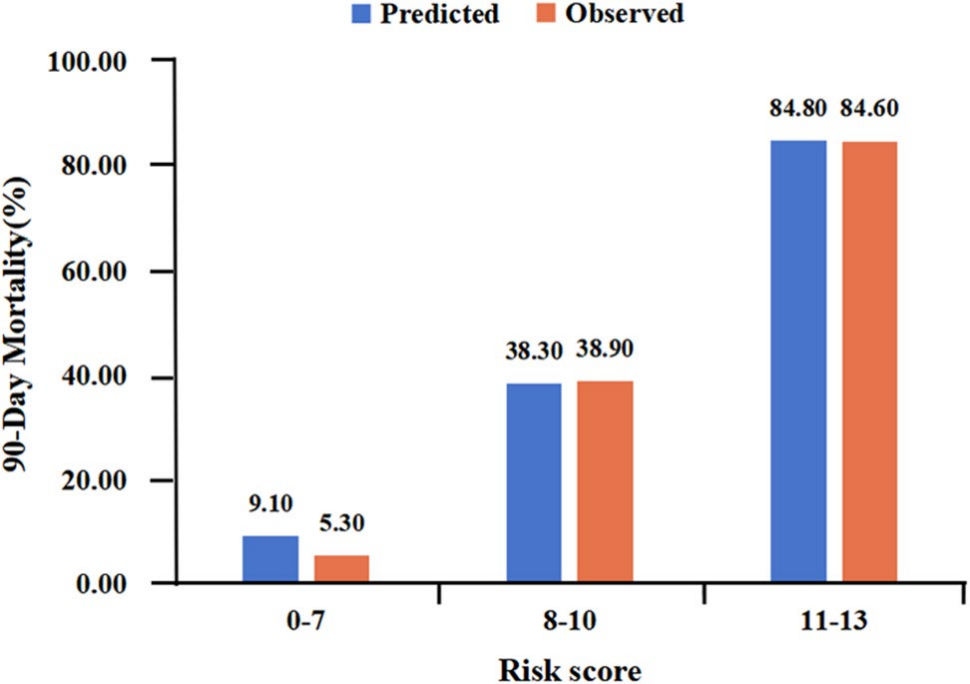
Observed versus predicted 90-day mortality among three groups in the test set

**Fig. 4:**
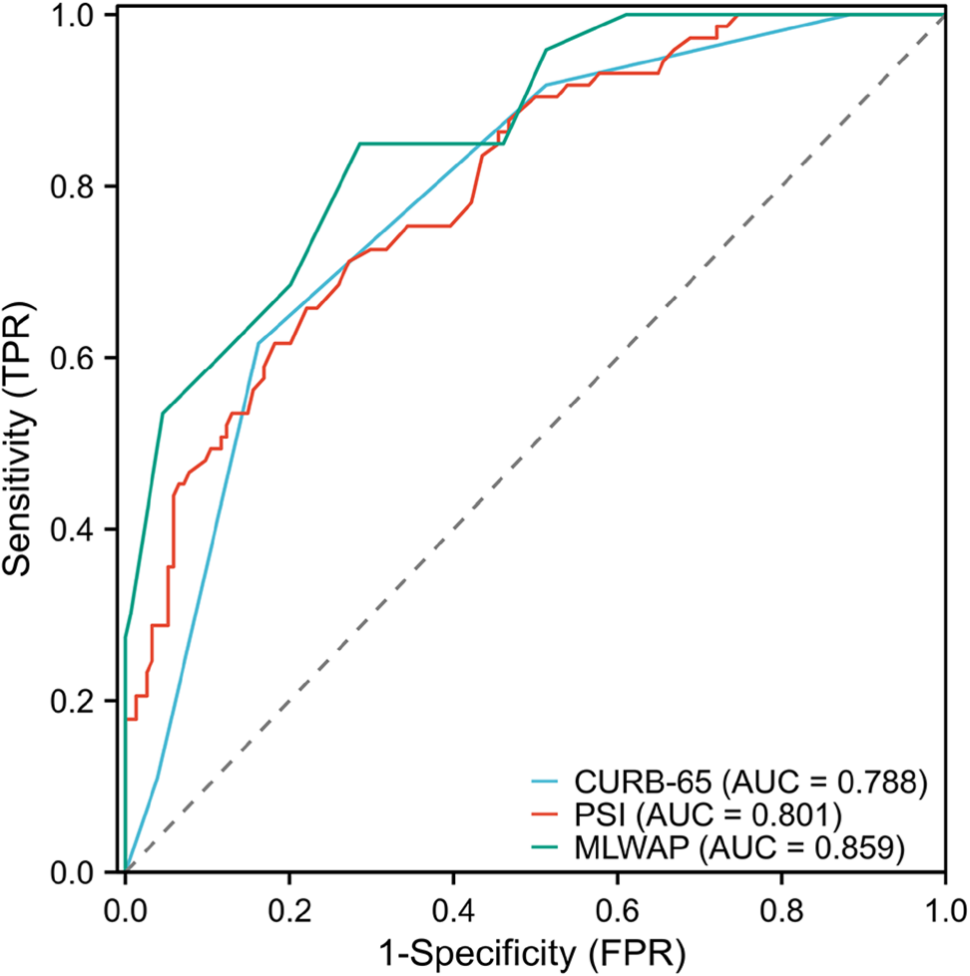
Characteristic curves for prediction of 90-day death of Omicron pneumonia patients (*n* = 227). AUROC of our MLWAP, CURB-65 and PSI score are 0.859, 0.788 and 0.801, separately

Based on the above methods and analysis, the following categorical variables were analyzed by backward stepwise logistic regression: age, cerebrovascular disease, immunosuppression, fever, chest tightness, respiratory tract secondary infection, myocardial injury, AKI, sivelestat treatment, ICU admission, invasive mechanical ventilation, Omicron pneumonia, PO_2_/FiO_2_, Lac, white blood cell count, neutrophil count, lymphocyte count, CRP, PCT, IL-6, and d-dimer. To develop a simple clinical prediction tool, we allocated relative weights based on the regression coefficients of each categorical variable (*β*). Table 2 shows the coefficients with the OR and 95% CI, and calculation of the immunosuppression, Lac ≥ 2.4, white blood cell count ≥ 6.7 × 10^9^/L, age ≥ 77, and PO_2_/FiO_2 _≤ 211 (MLWAP) scores. The OR generated by the regression analysis model was rounded to the nearest integer, resulting in a total score of 13, with immunosuppression, Lac ≥ 2.4, white blood cell count ≥ 6.7 × 10^9^/L, age ≥ 77 years, and PO_2_/FiO_2_ ≤ 211 given the respective scores of 5, 3, 2, 2, and 1 (Table 2). The points for each predictor were summed to calculate the total risk score for each patient. The AUC for the risk score model was 0.852 (95% CI, 0.792–0.912), similar to the AUC of the test set, 0.875 (95% CI, 0.789–0.961; Fig. 2a, b). The calibration curve showed a relatively good fit (Fig. 2c, d). We divided the risk score into low- (0–7 points; 90-day death risk < 30%), medium- (8–10 points; 90-day death risk 30–50%), and high- (11–13 points; 90-day death risk > 50%) risk groups (Figure S1). The incidence of predicted 90-day mortality with this model was similar to that observed in the test set (Fig. 3). This model had a sensitivity of 0.849, specificity of 0.714, and better predictive ability than the CURB-65 and PSI scores (AUROC = 0.859 *vs*. 0.788 *vs*. 0.801, respectively; Fig. 4).

**Table 2:** Final multivariable risk score and corresponding variables

Variables	Beta	S.E	Z	P	OR (95%CI)	Points
Immunosuppression	1.33	0.64	2.07	0.039	3.79 (1.07–13.38)	5
Lac ≥ 2.4	0.57	0.17	3.35	< 0.001	1.77 (1.27–2.47)	3
White blood cell counts ≥ 6.7 ∗ 10^9^ /L	0.19	0.07	2.67	0.008	1.21 (1.05–1.39)	2
Age ≥ 77	0.10	0.04	2.66	0.008	1.11 (1.03–1.19)	2
PO_2_/FiO_2_ ≤ 211	-0.01	0.00	-3.33	< 0.001	0.99 (0.98–0.99)	1

*Lac* lactate

## Discussion

The widespread transmission of COVID-19 is closely related to the continuous evolution of SARS-CoV-2 since its outbreak, which has raised enormous challenges for global epidemic prevention and control [17]. The Omicron variant has spread worldwide and has many mutations that affect its infectivity and immune escape [18]. The Chinese epidemic prevention and control policy was modified in December 2022, and the outbreak of the Omicron variant in China placed unprecedented pressure on the Chinese medical system. Few studies have examined the clinical characteristics and prognosis of Omicron pneumonia. In this context, we surveyed the clinical and epidemiological characteristics of older patients with Omicron pneumonia to develop a new score for predicting mortality risk. This score will facilitate early disease risk stratification and medical intervention to reduce mortality in critically ill patients.

This study investigated the clinical features of 227 older Omicron pneumonia patients who were treated in a tertiary hospital after the Chinese epidemic prevention and control policies were changed. The dominant variants in China at the time were Omicron BA 5.2 and BF.7. In our series, 32.2% (73/227) of the patients with Omicron pneumonia died, which is much greater than the 4.2% (10/238) that Xu et al. reported during the same period [19]. That study enrolled immunocompetent Omicron pneumonia patients with an average age of 71.84 ± 14.11 years, with comorbid cerebrovascular disease in 57.56% (137/238) and cardiovascular disease in 11.76% (28/238). Our study population comprised patients aged 60 years and older; the median age of the entire training set and its 90-day death group was 77 years and 81 years, respectively. Their leading comorbidity was cardiovascular disease (70.25%; 111/158), followed by immunosuppression (58.23%; 92/158). A previous study showed that older COVID-19 patients and patients with pre-existing disease develop more severe disease and have higher mortality rates [20]. Male gender, advanced age, and concomitant hypertension are associated with prolonged viral RNA shedding [21]. Moreover, age is positively associated with the severity and mortality of COVID-19 infection [11]. In addition, the large number of cases in a short period led to insufficient medical resources and ICU beds, delaying treatment in some severe and critical high-risk patients. Based on the 10th Chinese Novel Coronavirus infection diagnosis and treatment protocol, severe and critically ill high-risk patients are people (1) over 65 years old, especially those who have not been vaccinated with the COVID-19 vaccine; (2) with cardiovascular and cerebrovascular diseases, chronic lung diseases, diabetes, chronic liver and kidney diseases, tumors, and other comorbidities and maintenance dialysis; (3) immunodeficiency (such as AIDS, immunodeficiency caused by long-term use of corticosteroids or other immunosuppressive drugs); (4) BMI > 30 kg/m^2^; (5) late pregnancy and perinatal women; and (6) heavy smokers. Since our study population had many high-risk factors for developing severe and critical COVID-19, this may be the most crucial reason for the high mortality.

In our training set, the main symptoms were cough (113, 71.52%), fever (110, 69.62%), chest tightness (81, 51.27%), and pharyngalgia (17, 10.76%), which was similar to the study population in Miao et al. [22]. The CURB-65 and PSI scores in the 90-day death group were higher than in the survivor group (*p* < 0.001). As the scores most commonly used to evaluate disease severity and determine the appropriate nursing site for CAP, the CURB-65 and PSI scores are used in emergency departments and clinics for deciding the best setting for continuing patient management. Armiñanzas reported that the CURB-65 was an alternative score for predicting severity in patients with COVID-19 [23]. Another study showed that the mortality of COVID‐19 patients with PSI grades IV–V was significantly higher than that of patients with PSI grades I–III, reaching 32.1% (9/28) [24]. In addition, the PSI score of older COVID‐19 patients is higher than that of young and middle-aged patients, reflecting a higher risk of severe illness [25]. While the two scores may be unsuitable for predicting progress. In another prospective cohort study, 26.3% (CURB-65 score 0–2 points) and 37.7% (PSI grade I–III), with the development of ARDS, and both scores suggest low risk [26].

In the 90-day death group, the PO_2_/FiO_2_ and lymphocyte count were lower than in the survivor group (*p* < 0.001). PO_2_/FiO_2_ can be used as an index of the severity of respiratory insufficiency and the therapeutic effect. Franchini et al. found that PO_2_/FiO_2_ was the strongest determinant of survival in COVID-19 patients [27]. As an essential part of the adaptive immune system, lymphocytes play a vital role in eliminating invasive viruses and protecting human health from the threat of viruses. One study found that lymphopenia is a common characteristic of COVID-19 patients [28]. Moreover, Chen et al. found that natural killer (NK), CD4 + T, and CD8 + T cells in severely ill patients secreted less IFN-γ, suggesting that severe COVID-19 patients were immunosuppressed [29]. In the 90-day death group, the Lac content, white blood cell and neutrophil counts, CRP, PCT, IL-6, and d-dimer were all higher in the 90-day death group than in the survivor group (all *p* < 0.001), which was consistent with previous studies [19, 23] As a product of anaerobic metabolism, lactate is considered a powerful indicator for assessing the severity of sepsis [30]. Gupta et al. indicated that lactate is a key factor involved in the death of COVID-19 patients [31]. Neutrophilia is associated with a high inflammatory state and cytokine storm, a crucial part of the pathogenic mechanism of COVID-19 [32]. Soraya et al. found that the white blood cell and neutrophil counts were significantly higher in patients with severe COVID-19 than in non-severe cases, and increased with COVID-19 disease progression [33]. As an inflammatory biomarker, CRP is related to the development of COVID-19 and is an early predictor of severe disease [34]. IL-6, a vital inflammatory cytokine in the development of COVID-19, leads to acute lung injury, acute respiratory distress syndrome (ARDS), and further tissue damage [35]. One study showed that IL-6 ≥ 74.98 pg/mL, CRP ≥ 81 mg/L, PCT ≥ 0.56 ng/mL, and d-dimer ≥ 760 ng/mL effectively predicted the in-hospital mortality in COVID-19 patients [36].

In the training set, 26.58% (42/158) of the patients were admitted to the ICU, and 13.29% (21/158) received invasive mechanical ventilation. In the 90-day death group, 56.60% (30/53) were admitted to the ICU and 35.85% (19/53) received invasive mechanical ventilation, both more than in the survivor group (*p* < 0.05). This suggests that those Omicron pneumonia patients who died became critically ill quickly, needed ICU admission and invasive mechanical ventilation, and had a poor prognosis. A recent meta-analysis of 26 original studies and two systematic reviews including 83,619 COVID-19 patients reported an average ICU admission rate of 20.1% (range 4.6–32%); in the studies reporting ventilator support rates, an average of 48.8% needed invasive mechanical ventilation; moreover, the average mortality rate in the ICU was 34.9% (range 0–72%) [37]. Our findings were similar to those of Aziz et al., except that 13.66% of our entire sample required invasive mechanical ventilation versus 48.80% in Aziz et al. [37]. Possible reasons were that the number of Omicron pneumonia patients increased sharply, causing a shortage of medical resources; and some patients refused invasive mechanical ventilation for their own reasons.

In terms of complications, the complications in the 90-day death group included myocardial injury in 71.70% (38/53), AKI in 60.3% (32/53), and respiratory tract secondary infection in 45.28% (24/53), all significantly more frequent than in the survivor group (all *p* < 0.05). Zhou et al. reported that secondary infection decreases the survival of COVID-19 patients, especially those admitted to the ICU, and found that 15% (29/191) of COVID-19 patients had a secondary infection with 31% of these patients requiring mechanical ventilation in the ICU, and that 50% of the non-survivors had secondary infections versus only 1% of the survivors [38]. In another study, myocardial injury related to COVID-19 occurred in 15.0–27.8%, and was directly related to the risk of disease severity and death, increasing the risk of fatal events by 8–21 times [39]. A recent review revealed that about 31.5% of hospitalized COVID-19 patients developed AKI, and the death risk of COVID-19 patients with chronic kidney disease (CKD) stage 4–5 was 2.5 times than that of patients with normal renal function or CKD stage 1–2 [40]. Our results were consistent with the previous studies.

In the 90-day death group, 30.19% (16/53) of the patients were treated with sivelestat, which was significantly higher than the percentage in the survivor group (*p* < 0.05). However, there were no statistical differences in the use of antiviral agents, anticoagulation, baricitinib, tocilizumab, human immunoglobulin, or glucocorticoid between the two groups. ARDS is one of the most common fatal complications of COVID-19, and its occurrence and progression to death is critically dependent on neutrophil elastase. Sivelestat is a neutrophil elastase inhibitor (NEI) used to treat ALI/ARDS [41]. Clinical trials have shown that sivelestat can significantly reduce the non-mechanical ventilation time and improve the survival rate of ALI/ARDS patients with lung injury score (LIS) < 2.5, and improve the adverse prognosis of ALI/ARDS patients. However, the STRIVE study found that sivelestat cannot be used as an early intervention for patients with LIS > 2.5 [42, 43]. There is no evidence supporting the use of NEIs in ARDS induced by COVID-19. Although we did not use the Murray score [44] to evaluate lung injury, based on the data for the 90-day death group, 83.02% (44/53) of the patients had multi-lobar infiltration, for a value of 3, with a mean PO_2_/FiO_2_ of 96.00 (68.00–153.00) for a value of 4; the final value is obtained by dividing the aggregate value (= 7) by the number of components (= 2), strongly suggesting that the lung injury scores exceeded 2.5. Treatment with sivelestat did not provide benefits, although our sample was small. We expect future clinical trials to evaluate the efficacy of sivelestat in COVID-19 patients with a high risk of respiratory failure.

The CURB-65 score, which evaluates confusion, urea levels, respiratory rate, blood pressure, and age ≥ 65 years, was proposed by the British Thoracic Association in 2003 and is mainly used to evaluate the 30-day mortality risk of CAP [9]. PSI is a five-grade comprehensive score proposed in 1997 by Fine [16], covering 20 variables such as age, gender, complications, vital signs, and laboratory tests, that is mainly used to predict the death risk of community pneumonia within 6 weeks. Genc Yavuz reported that a CURB-65 ≥ 3 points and PSI ≥ grade IV show considerable predictive performance for the 30-day death risk of COVID-19 patients over 60 years old (AUC = 0.832 *vs*. 0.846) [45]. When there is a heavy emergency department burden, CURB-65 can be considered when following patients over 60 years old. Some scholars found that the CURB-65 had a poorer predictive ability in influenza A virus for older patients than for younger ones [46, 47]. In older adults, the mortality rate of viral diseases was more than twice that of young patients [48]. Our entire sample consisted of patients aged 60 years and older; in the training set, the median age of the 90-day death group was 81 years, which was higher than that of the survivor group (74 years). This suggests that if we used the CURB-65 to predict the death risk of this sample, its efficiency might be reduced. In addition, when there is a heavy emergency department burden, the PSI score is complicated to use as it involves many variables.

All the parameters in the MLWAP score are easy to obtain and all the tests can be performed in a hospital. The AUC for the model in the training and test sets was 0.852 (95% CI, 0.792–0.912) and 0.875 (95% CI, 0.789–0.961), respectively. Hosmer–Lemeshow goodness-of-fit test in the two sets showed that the *p*-values were 0.948 and 0.975 respectively (both *p* > 0.05), suggesting the calibration curves with good fit, and it showed good consistency in predicting the death risk and actual risk of older patients with Omicron pneumonia. This model had a sensitivity of 0.849, specificity of 0.714, and better predictive ability than the CURB-65 and PSI scores (AUROC = 0.859 vs. 0.788 vs. 0.801, respectively). In summary, the MLWAP score shows promise for predicting 90-day death in older Omicron pneumonia patients.

There are some limitations to our study. First, as a retrospective study, uncertain bias and confounding factors cannot be addressed, and patients with a high risk of death may reduce the maximum intervention measures or even refuse ICU treatment; the mortality prediction was retrospective by introducing biased data. Second, establishing a mortality risk score for predicting disease prognosis may be evaluated better in a larger multi-center sample, and our sample size was relatively small. Finally, the score should be verified using larger international datasets to improve its universality and robustness.

## Conclusion

In summary, the MLWAP score, based on five conventional indexes available in hospitals, was a good predictor of 90-day mortality in older Omicron pneumonia patients. This tool may help clinicians predict these patients’ admission prognoses, thus assisting in the allocation of medical resources and contributing to a reduction in their mortality.

## Funding source

This work was supported by the Medical, Health Science and Technology Innovation Project of Suzhou, China (grant no. SKY2021006); this work was also supported by the Competitive discipline lift project of the Second Affiliated Hospital of Soochow University (grant no. XKTJ-XK202007); this work was also supported by the science and technology project of Suzhou Hospital of Integrated Traditional Chineseand Western Medicine (grant no. YJ2022026).

### Supplementary Information

Below is the link to the electronic supplementary material.

Supplementary file1 (DOCX 295 KB)

## Data Availability

Data are available on request due to privacy/ethical restrictions.
