# Supplementary material for: Clinical features that predict the mortality risk in older patients with Omicron pneumonia: the MLWAP score
Source: Intern Emerg Med. 2023 Dec 16;19(2):465–75. doi: 10.1007/s11739-023-03506-2 (PMC10954909; doi:10.1007/s11739-023-03506-2)
Supplement: Supplementary file 1 — Supplementary file1 (DOCX 295 KB) [file 11739_2023_3506_MOESM1_ESM.docx]

**Fig. S1** Risk stratification score for predicting 90-day mortality of elderly Omicron pneumonia patients. The plot shows the calculation and the distribution of the points and the corresponding predicted risks; Lac, lactate


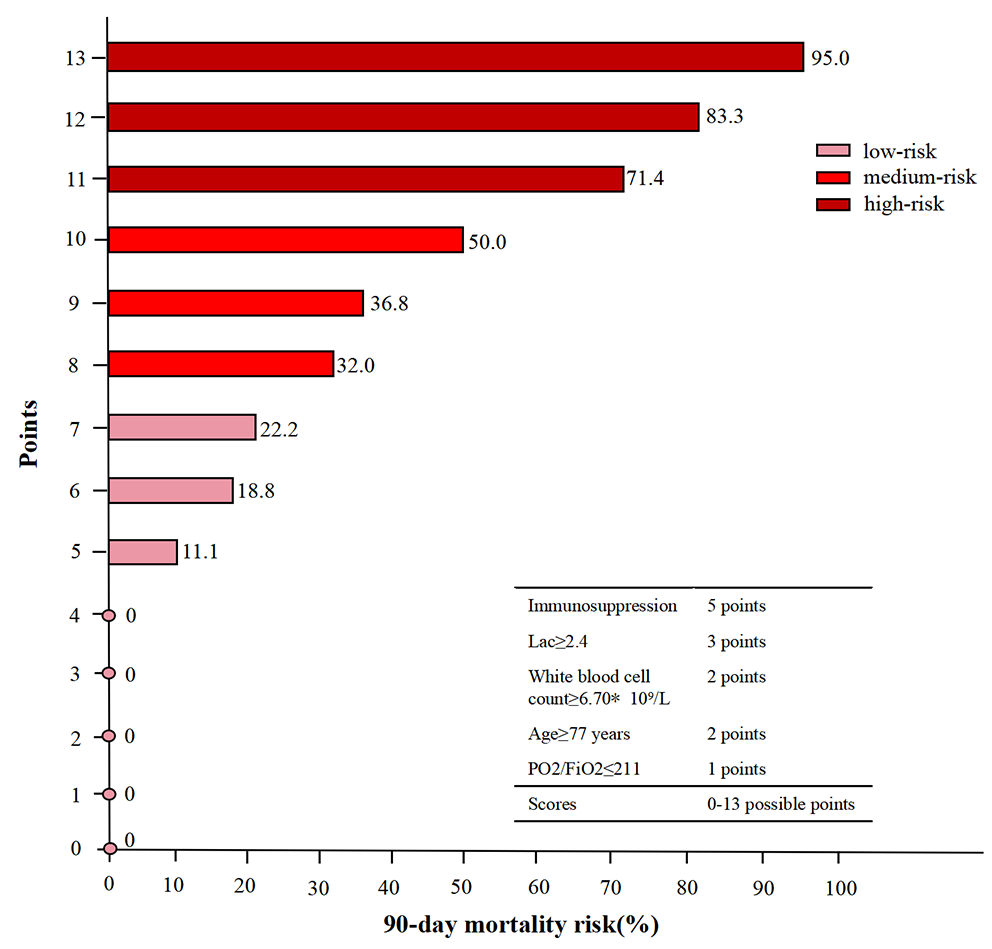


**Table S1** Comparisons of demographics and baseline characteristics between the training cohort and the validation cohort

| **Variable** | **Total (n = 227)** | **Training cohort**  **(n = 158)** | **Validation cohort**  **(n = 69)** | **χ2/Z** | ***P*** |
| --- | --- | --- | --- | --- | --- |
| male | 146 (64.32) | 102 (64.56) | 44 (63.77) | 0.013 | 0.909 |
| Age (years) | 77.00 (70.00 - 84.00) | 77.00 (70.00 - 84.00) | 77.00 (70.00 - 83.00) | 0.053 | 0.958 |
| BMI (kg/m^2^) | 23.00 (21.00 - 25.00) | 23.00 (21.00 - 25.00) | 23.00 (22.00 - 24.00) | 0.590 | 0.559 |
| chronic lung disease | 28 (12.33) | 17 (10.76) | 11 (15.94) | 1.193 | 0.275 |
| cardiovascular disease | 162 (71.37) | 111 (70.25) | 51 (73.91) | 0.315 | 0.575 |
| cerebrovascular disease | 32 (14.1) | 23 (14.56) | 9 (13.04) | 0.091 | 0.763 |
| diabetes | 77 (33.92) | 57 (36.08) | 20 (28.99) | 1.077 | 0.299 |
| immunosuppression | 132 (58.15) | 92 (58.23) | 40 (57.97) | 0.001 | 0.971 |
| malignant tumor | 47 (20.7) | 31 (19.62) | 16 (23.19) | 0.372 | 0.542 |
| Days of onset of symptoms before admission | 7.00 (4.00 - 10.00) | 7.00 (4.00 - 10.00) | 6.00 (3.00 - 9.00) | 1.561 | 0.123 |
| fever | 156 (68.72) | 110 (69.62) | 46 (66.67) | 0.195 | 0.659 |
| cough | 156 (68.72) | 113 (71.52) | 43 (62.32) | 1.891 | 0.169 |
| chest tightness | 117 (51.54) | 81 (51.27) | 36 (52.17) | 0.016 | 0.900 |
| pharyngalgia | 22 (9.69) | 17 (10.76) | 5 (7.25) | 0.677 | 0.411 |
| multi-lobular infiltration | 183 (80.62) | 129 (81.65) | 54 (78.26) | 0.352 | 0.553 |
| CURB-65 score | 2.00 (1.00 - 3.00) | 2.00 (1.00 - 3.00) | 2.00 (1.00 - 3.00) | 0.095 | 0.927 |
| PSI score | 107.00 (90.00 - 131.00) | 107.50 (91.00 - 128.00) | 107.00 (86.00 -133.00) | 0.012 | 0.990 |
| Omicron pneumonia |  |  |  | 1.787 | 0.409 |
| non-severe | 75 (33.04) | 48 (30.38) | 27 (39.13) |  |  |
| severe | 76 (33.48) | 54 (34.18) | 22 (31.88) |  |  |
| critical | 76 (33.48) | 56 (35.44) | 20 (28.99) |  |  |
| PO_2_/FiO_2_ | 215.00 (118.50 - 312.00) | 211.00(123.00 - 312.0) | 217.00(117.00-278.00) | 0.111 | 0.912 |
| Lac | 2.30 (1.50 - 4.00) | 2.40 (1.50 - 4.07) | 2.10 (1.40 - 3.70) | 0.797 | 0.426 |
| white blood cell counts, ×10^9^ /L [4–10] | 6.70 (4.60 - 9.70) | 6.70 (4.70 - 9.88) | 6.40 (4.30 - 9.30) | 0.667 | 0.505 |
| neutrophil counts, ×10^9^ /L [1.8–6.3] | 5.20 (3.35 - 8.50) | 5.20 (3.40 - 8.70) | 5.10 (3.20 - 8.40) | 0.332 | 0.740 |
| lymphocyte counts, ×10^9^ /L [1.1–3.2] | 0.70 (0.50 - 1.00) | 0.70 (0.50 - 1.00) | 0.70 (0.40 - 0.90) | 1.181 | 0.239 |
| CRP (mg/L) | 68.70 (23.75 - 141.95) | 63.85 (28.65 - 139.85) | 77.80 (20.10 - 144.60) | 0.226 | 0.821 |
| PCT(ng/mL) | 0.15 (0.06 - 0.66) | 0.15 (0.06 - 0.76) | 0.16 (0.06 - 0.39) | 0.365 | 0.715 |
| IL-6 (pg/mL) | 20.46 (4.32 - 58.13) | 20.23 (6.37 - 56.88) | 21.00 (3.45 - 61.31) | 0.510 | 0.610 |
| D- dimer (μg/mL) | 1.95 (0.83 - 5.99) | 2.15 (0.93 - 6.00) | 1.64 (0.74 - 5.96) | 1.131 | 0.258 |
| admitted to ICU | 63 (27.75) | 42 (26.58) | 21 (30.43) | 0.355 | 0.551 |
| invasive mechanical ventilation | 31 (13.66) | 21 (13.29) | 10 (14.49) | 0.059 | 0.808 |
| respiratory tract secondary infection | 58 (25.55) | 40 (25.32) | 18 (26.09) | 0.015 | 0.903 |
| myocardial injury | 99 (43.61) | 67 (42.41) | 32 (46.38) | 0.308 | 0.579 |
| AKI | 75 (33.04) | 53 (33.54) | 22 (31.88) | 0.060 | 0.807 |
| hepatic insufficiency | 106 (46.70) | 76 (48.10) | 30 (43.48) | 0.412 | 0.521 |
| antiviral | 143 (63.00) | 99 (62.66) | 44 (63.77) | 0.025 | 0.873 |
| sivelestat | 30 (13.22) | 23 (14.56) | 7 (10.14) | 0.815 | 0.367 |
| baricitinib | 18 (7.93) | 11 (6.96) | 7 (10.14) | 0.666 | 0.414 |
| tocilizumab | 14 (6.17) | 10 (6.33) | 4 (5.80) | 0.000 | ＞0.999 |
| human immunoglobulin | 40 (17.62) | 25 (15.82) | 15 (21.74) | 1.158 | 0.282 |
| glucocorticoid | 199 (87.67) | 143 (90.51) | 56 (81.16) | 3.880 | **0.049** |

Continuous variables presented as median (upper and lower quartiles), enumeration data as n (%). All clinical characteristics were collected on admission. *P*-value represented the comparison between the training cohort and the validation cohort. The bolded values are *p*-values < 0.05, which indicates the significant difference between the two cohorts. BMI, body mass index; Lac, lactate; COVID, coronavirus disease; CRP, C-reactive protein; PCT, procalcitonin; IL-6, Interleukin 6; ICU, intensive care unit; AKI, acute kidney injury.
